# Cortical excitability in patients with REM sleep behavior disorder with abnormal TRODAT-1 SPECT scan: an insight into prodromal Parkinson’s disease

**DOI:** 10.3389/fneur.2023.1156041

**Published:** 2023-05-24

**Authors:** Siao-Chu Su, Rou-Shayn Chen, Yi-Chieh Chen, Yi-Hsin Weng, June Hung, Yi-Ying Lin

**Affiliations:** ^1^Division of Movement Disorders, Department of Neurology, Linkou Chang Gung Memorial Hospital, Taoyuan, Taiwan; ^2^Neuroscience Research Center, Chang Gung Memorial Hospital, Taoyuan, Taiwan; ^3^Department of Neurology, Tucheng Hospital, New Taipei City, Taiwan; ^4^College of Medicine, Chang Gung University, Taoyuan, Taiwan

**Keywords:** REM sleep behavior disorder, cortical excitability, transcranial magnetic stimulation, input–output curve, short interval intracortical inhibition, silence period, TRODAT-1 SPECT

## Abstract

**Introduction:**

REM Sleep Behavior Disorder (RBD) has been highlighted to identify a patient with prodromal Parkinson’s disease (PD). Although many studies focus on biomarkers to predict an RBD patient’s evolution from prodromal PD to clinical PD, the neurophysiological perturbation of cortical excitability has not yet been well elucidated. Moreover, no study describes the difference between RBD with and without abnormal TRODAT-1 SPECT.

**Methods:**

By measuring the amplitude of motor evoked potentials (MEP), the cortical excitability changes after transcranial magnetic stimulation (TMS) were evaluated in 14 patients with RBD and eight healthy controls (HC). Seven of the 14 patients with RBD showed abnormal TRODAT-1 (TRA-RBD), and seven were normal (TRN-RBD). The tested parameters of cortical excitability include resting motor threshold (RMT), active motor threshold (AMT), short-interval intracortical inhibition (SICI), intracortical facilitation (ICF), contralateral silence period (CSP), and input–output recruitment curve.

**Results:**

The RMT and AMT showed no difference among the three studied groups. There was only SICI at inter-stimuli-interval 3 ms revealing group differences. The TRA-RBD demonstrated significant differences to HC in these aspects: decreased SICI, increased ICF, shortening of CSP, and augmented MEP amplitude at 100% RMT. Moreover, the TRA-RBD had a smaller MEP facilitation ratio at 50% and 100% of maximal voluntary contraction when compared to TRN-RBD. The TRN-RBD did not present any difference to HC.

**Conclusion:**

We showed that TRA-RBD shared similar cortical excitability changes with clinical PD. These findings would provide further insight into the concept that RBD is the highly prevalent entity in prodromal PD.

## 1. Introduction

Parkinson’s disease (PD) is the most prevalent α-synuclein-mediated neurodegenerative disorder. α-synucleinopathies include PD, Dementia with Lewy Bodies (DLB), and Multiple System Atrophy (MSA). PD is a slowly progressive disease and does not start acutely. This implies that PD advances from early phases when the degenerative process has commenced to the point where a PD patient can finally be diagnosed, and medical intervention can be initiated (1). While there is no curative treatment in PD, the physicians’ armamentarium of treatment strategies is symptomatic therapy. The initiation of symptomatic therapy is an essential milestone in disease progression (2). With such a perspective, efforts have been made to identify reliable biomarkers for earlier intervention to enhance the likelihood of success in modifying PD disease progression. The Parkinson’s Progression Markers Initiative (PPMI) established a PD biomarker cohort that extensively surveyed the clinical, imaging, genetic, and biospecimen of Parkinson’s disease to approach this goal with many publications since 2011 (3, 4). However, the neurophysiological changes have not been clearly elucidated in PPMI. It is interesting to investigate the alterations in the neural activity of cortico-basal ganglia motor loops, leading to dysfunctional motor output in patients with RBD when the clinical symptoms of PD have not been identified.

With increasing knowledge about neurodegenerative disorders, the International Parkinson and Movement Disorder Society (IPMDS) Task Force revised the conceptualizations and proposed that early PD should be divided into three stages: preclinical PD (neurodegenerative processes have begun, but there are no evident symptoms or signs), prodromal PD (although the symptoms and signs are noted, they are still insufficient to diagnose disease), and clinical PD (diagnosis could be made base on the proposed criteria) (5, 6). The proposed diagnostic criteria of prodromal PD highlighted the importance of identifying REM sleep behavior disorder (RBD) (6). The positive Likelihood ratio (LRs) was 130 in polysomnography-proven RBD and 2.3 in screen questionnaires diagnosed RBD (6). The prevalence of RBD in the general population is approximately 4.9% and can vary anywhere from 20% to 72% in PD patients (7). Furthermore, in patients with idiopathic RBD (iRBD), 40%–66% of subjects will develop parkinsonian syndrome within 10 years and 20 years, respectively (8, 9). The mean duration is between 3.7 and 7 years from the onset of RBD to the development of neurodegenerative disease (10). Therefore, from a clinical point of view, RBD carries a high risk of evolving into clinical PD. It would be interesting to identify if the cortical excitability changes in RBD shared similar characteristics with clinical PD to provide a possible explanation for why iRBD is the most frequent and important prodromal symptom of PD from the neurophysiological point of view.

Transcranial magnetic stimulation (TMS) is a well-validated non-invasive method to study cortical excitability, which is mandatory to evaluate the functional status of motor cortex circuity (11, 12). The reports in clinical PD documented that the abnormal cortical excitability preferentially contributed to the GABAergic circuit. In clinical PD, TMS studies revealed decreased short-interval intracortical inhibition (SICI) even in patients with imperceptible motor signs (13) and shortened silence periods (SP) (14). In patients with iRBD, the cortical excitability showed decreasing SICI and intracortical facilitation (ICF) intensity (15, 16). However, limited reports compared the difference between clinical PD patients with and without RBD (17, 18), and the cortical excitability changes in prodromal PD have not yet been well elucidated.

Tc-99 m TRODAT-1 SPECT is robust in identifying the dysregulation of central dopaminergic neurotransmission and would be a potential biomarker to predict an α-synucleinopathy (19). Nonetheless, abnormal TRODAT-1 SPECT is not essential to diagnosing clinical PD and iRBD (1). The TRODAT-1 could identify the reduction of striatum dopamine transporter (DAT) binding capacity to support the clinical PD diagnosis, but the sensitivity and specificity were not 100% (20, 21). The PPMI study reported that 36.8% of patients with iRBD will be clinically diagnosed with α-synucleinopathy after 4 years of follow-up (22). From these perspectives, abnormal TRODAT SPECT indicated the abnormal striatal uptake pathway but would not be enough to completely predict a patient on the way to manifesting clinical PD. Supposing the cortical excitability changes in RBD patients with abnormal TRODAT scans (TRA-RBD) have similar alterations with clinical PD, it will provide further insight to consider a patient with prodromal PD.

In our study, we proposed that patients with TRA-RBD would have more similarity of cortical excitability changes with clinical PD patients, and the patients with RBD but without abnormal TRODAT-1 (TRN-RBD) might present subtle changes. To approach this aim, we conducted this comprehensive TMS study. We assumed that in the RBD-driven diagnosis of prodromal PD, the disease progression would more likely be a continuum from TRN-RBD to TRA-RBD and finally to clinical PD.

## 2. Materials and methods

### 2.1. Subjects

The subjects were recruited from outpatient clinics in a tertiary medical hospital according to the following criteria: (1) repeated episodes of dream enactment behavior, including sleep-related vocalization or complex motor behaviors confirmed by the family or patients themselves, (2) completing the REM Sleep Behavior Disorder Screening Questionnaire (RBDSQ), (3) no evident motor symptoms to fulfill the diagnosis criteria of clinical PD or other parkinsonian syndromes, and (4) must have accepted the TRODAT-1 SPECT neuroimaging study. Healthy controls (HC) were recruited from a departmental register of volunteers.

All participants were not under the age of 20 years old. The exclusion criteria were a history of nervous system diseases (such as epilepsy, cerebrovascular diseases, and subdural effusion), psychiatric disorders (such as major depressive, bipolar, or schizoaffective disorders), head trauma, and medication history of antipsychotics, antidepressants, and illicit drugs use. Subjects who ever received intracranial operation and a person with alcohol use disorder were also excluded.

All subjects provided informed consent before participation in this study. The study was performed in accordance with the Declaration of Helsinki and was reviewed and approved by the Chang Gung Memorial Hospital institutional review board (IRB number: 202200708A3).

### 2.2. TRODAT-1 SPECT

**Figure 1:**
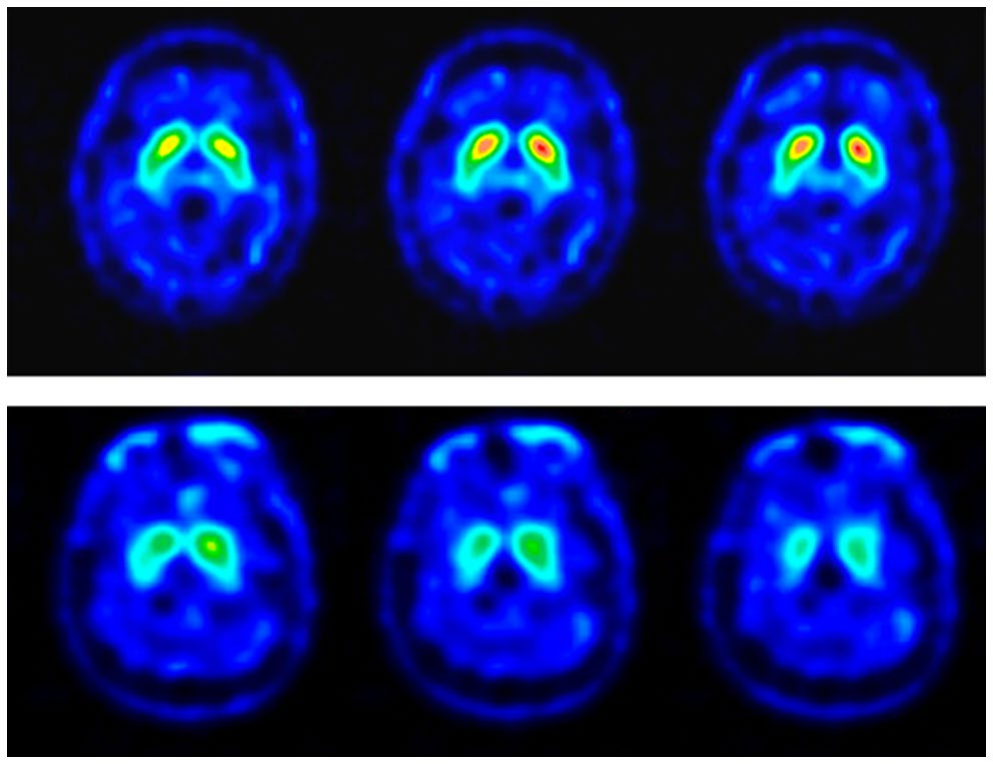
TRODAT-1 SPECT images of the RBD patients. The color scale represents the specific bindings to the dopamine re-uptake transporter in different levels of the striatum. The images in the upper row are the SPECT images of a 64-year-old male patient with no definite presynaptic dopaminergic lesion (TRN-RBD). The lower row showed the SPECT images of a 49-year-old female patient with a uniform pattern of radioactivity loss in the striatum which is possibly more dominant over the right side (TRA-RBD).

The dopamine transporter brain image was performed at the Nuclear Medicine Department. The SPECT images were acquired 4 h after 25 mCi of Tc-99 m TRODAT was given intravenously. A triple-head camera with fan beam collimators was used for the SPECT images, with 120 equally spaced projections over a 360° arc acquired in a 128 × 128 matrix size. Individual images were reconstructed with back-projection using a ramp–Butterworth filter for the effects of photon attenuation, and the attenuation correction was done using Chang’s first-order method. Three reconstructed transaxial slices were summed together, paralleling the orbitomeatal line with the highest signal in the basal ganglia region as the central slice. After evaluating the radioactivity uptake intensity and distribution, experienced nuclear physicians double-checked and reported all images. The examples of TRODAT-1 SPECT images are demonstrated in Figure 1.

### 2.3. TMS

During the experiments, subjects were seated in comfortable chairs. Electromyographic (EMG) recordings were made using surface Ag–AgCl electrodes from the target muscles. The EMG signal was amplified (Digitimer D360; Wel-wyn Garden City, Hertfordshire, United Kingdom), bandpassed (3 Hz–2 kHz), and analyzed offline on a personal computer. In the TMS experiments, EMG signals were recorded from the contralateral first dorsal interosseous (FDI) with a gain of 5,000 and 1,000. Magnetic stimulation was given using a handheld double 70 mm Coil (Magstim Co., Whitland, Dyfed, United Kingdom) connected through a Bistim module (Magstim Co.) to two Magstim 200 machines (Magstim Co.). The coil was positioned tangentially to the scalp, with the handle pointing backward. The dominant hemisphere was studied in all subjects. We defined the location of the “motor hot spot” of the hand area as the location on the scalp where magnetic stimulation produced the largest motor-evoked potential (MEP) from the contralateral FDI when the subject was relaxed. An oscilloscope continuously monitored the surface EMG of the FDI throughout the experiments to ensure complete muscle relaxation in all subjects.

#### 2.3.1. Motor threshold

The resting motor threshold (RMT) was defined as the minimum stimulation intensity over the hot spot that could elicit a motor-evoked potential (MEP) of not less than 50 μV in five out of 10 trials. The active motor threshold (AMT) was defined as the minimum stimulation intensity over the motor hot spot that could elicit an MEP of not less than 200 μV in five out of 10 trials during a voluntary contraction of the contralateral FDI.

#### 2.3.2. Input–output (I/O) curve

Single-pulse TMS was given in three blocks at three different intensities: 100%, 110%, and 120% of RMT. Ten stimuli at intervals of 5 s were delivered in each block. The three blocks were conducted in a random sequence. Moreover, Single-pulse TMS was also given in three blocks at three different intensities of voluntary muscular contraction: relaxation, 50% of maximal voluntary contraction (MVC), and 100% MVC at the TMS 110% of RMT in the RBD patients. Ten stimuli at intervals of 5 s were delivered in each block. The peak-to-peak amplitude of MEPs was measured and averaged offline for each intensity.

#### 2.3.3. Paired-pulse stimulants

In this research, the subthreshold conditioning stimulus was given at 80% AMT, and the test stimulus was given at the intensity required to produce an MEP of 1 mV for the test of short interval intracortical inhibition (SICI) and intracortical facilitation (ICF). The SICI is the reduction of MEP at short interstimulus intervals (ISIs 1–4 ms), while ICF is the effect on MEP under longer ISIs of between 8 and 15 ms. Subjects received in random order either the test stimulus alone or conditioning-test stimuli at ISIs of 2, 3, 4, 7, and 10 ms for 10 trials per condition. All trials in which EMG movement artifacts occurred were rejected online and that stimulus condition was repeated.

#### 2.3.4. Silent period (SP)

Subjects were asked to squeeze a 5-cm block between the thumb and index finger. Visual feedback on the intensity of muscle contraction was provided to the subjects, and they were instructed to maintain a constant muscle contraction at ~20% of the maximum. Magnetic stimulation was applied over the contralateral hand motor area at an intensity of 120% RMT. Fifteen stimuli were recorded for each subject. The SP was calculated by measuring the time from the end of the MEP to the reappearance of continuous EMG activity over 20 μV. Trials in which voluntary muscle activation exceeded or was less than 20% of the maximum were rejected online and the stimulus was repeated.

### 2.4. Statistical analysis

All the data were analyzed by the IBM SPSS Statistics 19 software, and the level of *value of p* significance was set at 0.05. The results were presented as mean ± standard deviation (SD). Statistical analysis of the variations of TMS parameters including the RMT, AMT, SICI, ICF, SP, and MEP amplitude was performed by non-parametric Kruskal-Wallis ANOVA. Mann–Whitney U test was used for the *post hoc* analysis and the comparison between each pair of groups of the demographic data (disease onset age, disease duration, UPDRS part III, and RBDSQ score), and all the TMS parameters were measured.

## 3. Results

### 3.1. Demographic and clinical data

Fourteen patients with RBD and eight healthy controls completed the TMS examination smoothly, without adverse effects reported. Table 1 summarizes the clinical demographic characteristics of the patients. All the RBD patients had neither clinically evident motor symptoms nor associated disorders. The possibility of prodromal PD could be considered even though the Unified Parkinson’s Disease Rating Scale (UPDRS) ranged from 0 to 12. As an illustration in Figure 1, seven patients showed abnormal TRODAT-1 SPECT (TRA-RBD group) and seven were normal (TRN-RBD group). There were no significant differences between the two RBD groups in the disease duration, RBDSQ score, and UPDRS Part III score.

**Table 1:** Demographic characteristics of patients with RBD.

Assessment	TRA-RBD (*n* = 7)	TRN-RBD (*n* = 7)	Mann–Whitney U test *p-*value
Onset age (years)	56.6 ± 8.9	50.9 ± 11.9	0.480
Disease duration (years)	7.43 ± 4.392	11.71 ± 9.178	0.456
RBDSQ	7.43 ± 3.457	8.43 ± 3.101	0.535
UPDRS Part III score	7.43 ± 3.599	3.86 ± 2.193	0.073

There is no statistical significance between all items of assessment. *n*, patient number; RBD, REM sleep behavior disorder; TRA-RBD, patients with RBD and abnormal TRODAT-1 SPECT; TRN-RBD, patients with RBD and normal TRODAT-1 SPECT; RBDSQ, REM Sleep Behavior Disorder Screening Questionnaire; UPDRS, Unified Parkinson’s Disease Rating Scale.

### 3.2. Motor thresholds and input–output (I/O) curves

Table 2 discloses the single-pulse and paired-pulse TMS parameters among all the participants. The RMT and AMT cannot differentiate between the TRA-RBD, TRN-RBD, and HC.

**Table 2:** Single and paired-pulsed TMS comparison between patient groups and HC.

Examination	TRA-RBD (*n* = 7)	TRN-RBD (*n* = 7)	HC (*n* = 8)	Mann–Whitney U test *p-*value			Kruskal Wallis Test *p-*value
				TRA vs. TRN	TRA vs. HC	TRN vs. HC	
RMT (%)	62.7 ± 10.0	69.0 ± 11.05	65.88 ± 14.317	0.318	0.817	0.685	0.647
AMT (%)	48.4 ± 12.2	55.1 ± 13.2	44.25 ± 9.362	0.128	0.485	0.131	0.179
MEP amplitude (mV)							
Rest, 100% RMT	0.23 ± 0.22	0.09 ± 0.09	0.10 ± 0.06	0.085	0.064	0.418	0.100
Rest, 110% RMT	0.45 ± 0.53	0.33 ± 0.42	0.371 ± 0.24	0.277	0.643	0.487	0.584
50% MVC, 110% RMT	1.28 ± 0.78	3.33 ± 2.63	n.a.	0.142	n.a.	n.a.	n.a.
100% MVC, 110% RMT	2.43 ± 2.13	5.28 ± 3.04	n.a.	0.142	n.a.	n.a.	n.a.
SICI ratio (ISI 3 ms, %)	98.6 ± 28.3^*,†^	70.2 ± 32.3^†^	53.4 ± 10.9^†^	0.064	0.008	0.105	0.012
ICF ratio (ISI 10 ms, %)	168.6 ± 76.2^*^	180.2 ± 118.9	112.9 ± 16.2	0.565	0.037	0.247	0.126
CSP (ms)	91.6 ± 20.1^*^	109.9 ± 18.5	120.1 ± 14.4	0.18	0.028	0.298	0.071

*n*, patient number; n.a., not available; HC, healthy controls; RBD, REM sleep behavior disorder; TRA-RBD, patients with RBD and abnormal TRODAT-1 SPECT; TRN-RBD, patients with RBD and normal TRODAT-1 SPECT; RMT, resting motor threshold; AMT, active motor threshold; MEP, motor-evoked potentials; MVC, maximal voluntary contraction; SICI, short interval intracortical inhibition; ICF, intracortical facilitation; CSP, contralateral silence period. ^*^Significant difference between TRA-RBD and HC (Mann–Whitney U test; *p* < 0.05). ^†^Significant difference among the three groups (Kruskal Wallis Test; *p* < 0.05).

**Figure 2:**
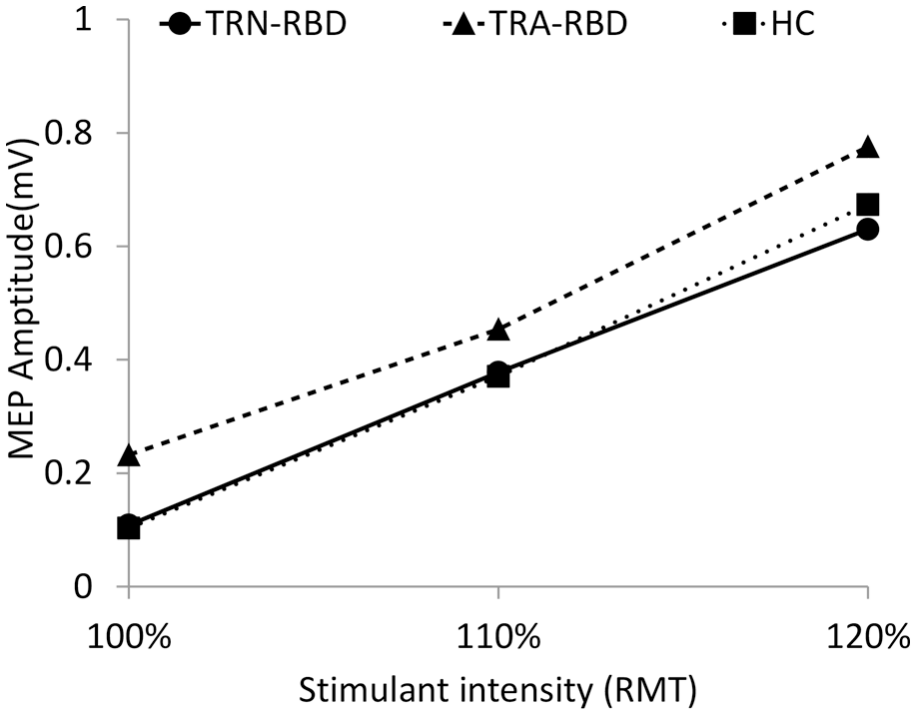
I/O curve at different stimulant intensities during resting. This figure demonstrates the MEP amplitude during different stimulant intensities. The X-axis indicates the percentage of RMT, and the Y-axis shows the mean peak-to-peak MEP amplitude. The volume of amplitude and the *p*-values of statistical analysis are listed in Table 2. There was also no group significance in the 120% RMT analyzed by the Kruskal Wallis Test (*p* = 0.555).

Figure 2 illustrates the amplitude and curve steepness of either the RBD group or the HC after increasing TMS stimulating intensity. The slopes of the I/O curves were similar, showing no group difference. The baseline MEP amplitude at 100% RMT was larger in TRA-RMD than in TRN-RBD and HC. However, the statistical differences were borderline; the *p*-value was 0.085 between TRA-RBD and TRN-RBD and 0.064 between TRA-RBD and HC.

**Figure 3:**
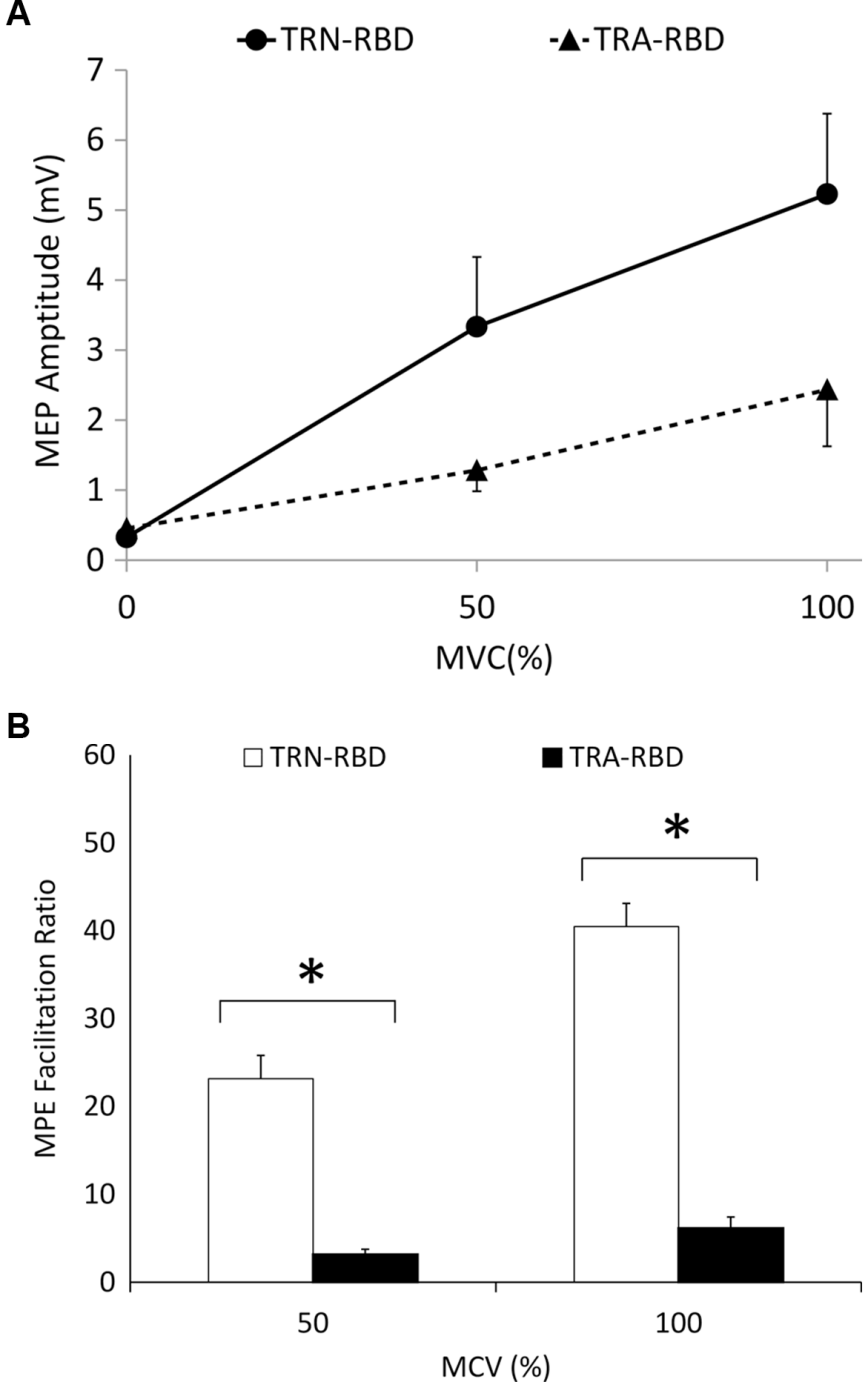
**(A)** I/O curve during voluntary contraction. This figure demonstrates the MEP amplitude during different MVC at the stimulant intensity of 110% RMT. The X-axis indicates the percentage of MVC, and the Y-axis shows the mean peak-to-peak MEP amplitude. The standard error is shown as the error bar. There was no statistical significance of the amplitude or the steepness. The *p*-value between the steepness of MVC 0%–50%, 50%–100%, and 0%–100% were 0.085, 0.180, and 0.064, respectively. The *p*-values between the amplitude are disclosed in Table 2. **(B)** Voluntary contraction facilitation ratio of MEP. The facilitation ratio is the MEP amplitude during involuntary contraction divided by the resting MEP amplitude. This figure shows the facilitation ratio of the MEP volume during different MVC at the stimulant intensity of 110% RMT. The reduced facilitation ratio was found in TRA-RBD patients compared with TRN-RBD patients at both MVC 50% (*p* = 0.048) and MVC100% MVC (*p* = 0.013). *Significant difference between TRA-RBD and TRN-RBD (Mann–Whitney U test; *p* < 0.05).

Figure 3 demonstrates the amplitude and curve steepness between TRA-RBD and TRN-RBD by increasing the MCV. There was no difference in the absolute MEP amplitude between the two groups at the contraction strength of 50% and 100% MCV. However, the MEP amplitude ratio revealed a significant reduction at either MCV.

### 3.3. Short intracortical inhibition and intracortical facilitation

**Figure 4:**
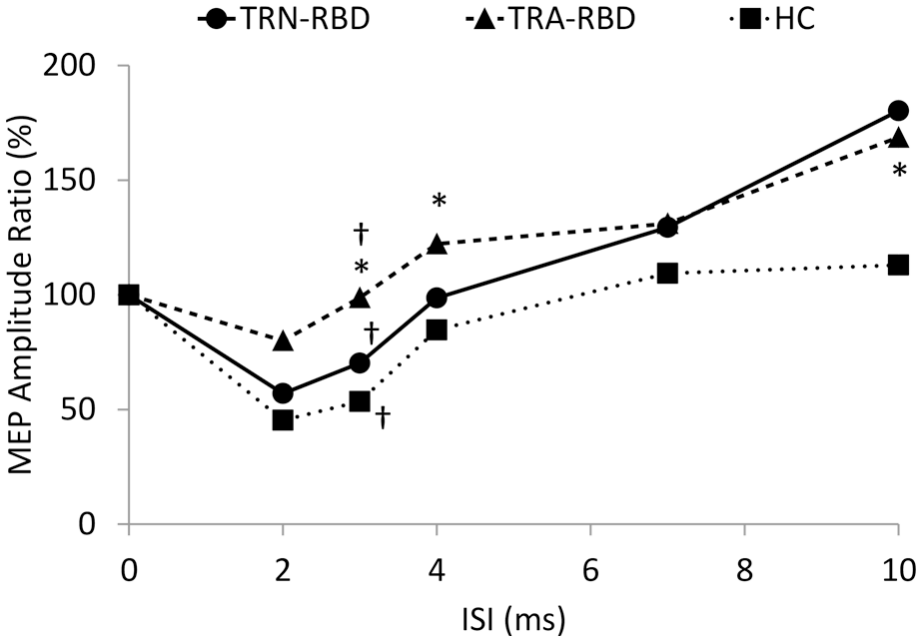
Paired pulse stimulation. This figure demonstrates the MEP amplitude ratio with different conditioning ISI. The X-axis shows the different ISI between the conditioning and test stimuli. The Y axis shows the ratio of the mean peak-to-peak MEP amplitude to unconditional MEP. The decreased SICI was noted at ISI 3 ms (*p* = 0.008) and ISI 4 ms (*p* = 0.001) in the TRA-RBD compared with HC. Mild enlargement of ICF at ISI 10 ms was noted in the TRA-RBD compared to HC (*p* = 0.037). The group difference among TRA-RBD, TRN-RBD, and HC was found at ISI 3 ms (*p* = 0.012). *Significant difference between TRA-RBD and HC (Mann–Whitney U test; *p* < 0.05); †Significant difference among the three groups (Kruskal Wallis Test; *p* < 0.05).

The MEP amplitude ratio of the paired-pulse study is demonstrated in Figure 4. The Kruskal Willis examination identified the group difference among TRA-RBD, TRN-RBD, and HC at 3 ms (*p* = 0.012). In the TRA-RBD, the reduction of SICI was significantly different from the HC at ISI 3 ms and 4 ms (*p* = 0.008 and *p* = 0.011, respectively) but not different from the group of TRN-RBD. There was no difference in the SICI between TRA-RBD and TRN-RBD although the mean value of the SICI at ISI 3 ms was 98.6% in TRA-RBD and 70.2% in TRN-RBD. The intracortical facilitation of ICF was significantly enhanced in the TRA-RBD at an ISI of 10 ms when compared with the HC but not with the TRN-RBD. It should be noted that the mean value of the MEP ratio of TRN-RBD in Figure 4 looked ever larger than TRA-RBD; however, the non-significance between TRN-RBD and HC might be the limitation of small sample size and wide standard deviation.

### 3.4. Silent period

The period of SP was 120.1 ± 14.4 in HC, 109.8 ± 18.4 in TRN-RBD, and 109.8 ± 18.4 in TRA-RBD. Although there was a trend of advanced shortening of the SP, the Kruskal Wallis statistical analysis did not show the group difference. In the subgroup analysis, there was a significant difference between TRA-RBD and HC (*p* = 0.028) but not between the TRN-RBD and HC, as well as between the TRA-RBD and TRN-RBD.

## 4. Discussion

We documented TMS-driven cortical excitability changes over the primary motor cortex in patients with RBD. To our best knowledge, this study would be the first to compare these changes between RBD patients with and without abnormal TRODAT-1 SPECT. Our data showed the TRA-RBD had more extensive abnormality than healthy subjects in the following ways: (1) There was a trend of higher enhancement of MEP amplitude with increasing TMS intensity (Figure 2). (2) The MEP amplitude ratio showed a significant reduction when increasing voluntary muscular contraction (Figure 3). (3) The SICI was significantly decreased (Figure 4). (4) The silent period was shorter than in the healthy control (HC). While these parameters expressed no significant difference between TRN-RBD and HC, our results support the hypothesis that the TRA-RBD shares more cortical excitability alternations with PD compared to TRN-RBD or HC.

The cortical excitability changes have been a long-term research topic on iRBD and PD (Table 3); nonetheless, seldom studies reported fully comprehensive parameters but focused on specific variables to explain different issues (23). Most studies compared the cortical excitability between patients with iRBD and HC but did not analyze the iRBD subgroup (15, 16). Although the conclusions were not homogenous, the essential findings in patients with RBD were reduced ICF and equivocally decreased SICI. In patients with PD, the well-accepted concepts were in the order of decreased SICI, shortened SP, preserved or decreased ICF, and abnormal recruitment I/O curves by increasing TMS stimulation intensity or maximal voluntary contraction (MVC) power.

**Table 3:** Brief summary of studies of transcranial magnetic stimulation in RBD and PD.

		RMT	SICI	ICF	SP	I/O curve Slop	MEP facilitation	Ref.
Comparison between idiopathic RBD and HC								
1	Nardone et al.	–	–	–	n.a.	n.a.	n.a	(23)
2	Lanza et al.	–	–	↓	–	n.a.	n.a.	(15)
Comparison between PD with RBD and HC								
3	Lanza et al.	–	–	↓	–	n.a.	n.a.	(16)
Comparison among PD with RBD, PD without RBD, and HC								
4	Nardone et al.	–	–	–	–	n.a.	n.a.	(17)
5	Bhattacharya et al.	–	↓^*^	↓^†^	–	n.a.	n.a.	(18)
Comparison between PD and HC								
6	Cantello et al.	↓	n.a.	n.a.	↓	↑	↓	(29)
7	Valls-Sole et al.	–	n.a.	n.a.	↓	↑	↓	(24)
8	Priori et al.	–	n.a.	n.a.	↓	n.a.	n.a.	(40)
9	Nakashima et al.	–	n.a.	n.a.	↓	n.a.	n.a.	(41)
10	Strafella et al.	–	↓	–	n.a.	n.a.	n.a.	(33)
11	Pierantozzi et al.	–	↓	n.a.	↓	n.a.	n.a.	(34)
12	Lou et al.	↓	n.a.	n.a.	n.a.	n.a.	↑	(28)
13	Baresˇ et al.	–	↓	–	n.a.	n.a.	n.a.	(35)
14	MacKinnon et al.	–	↓	n.a.	n.a.	n.a.	n.a.	(36)
15	Ni et al.	–	↓	n.a.	n.a.	n.a.	n.a.	(37)
16	Bologna et al.	–	–	–	n.a.	↑	n.a.	(25)
17	Guerra et al.	–	–	–	n.a.	↑	n.a.	(26)
18	Ammann et al.	–	↓	–	n.a.	n.a.	n.a.	(13)

PD, Parkinson’s disease; Ref., reference number; n.a., not available; The other abbreviations are identical to Table 2. I/O curve slop, the steepness of the curves’ slope; MEP facilitation, the intensity of MEP amplitude after increasing voluntary muscular contraction. ↓, decreased; ↑, increased; –, no difference in the study group; ↓^*^, only decreased in PD with RBD not in PD without RBD; ↓^†^, decreased in PD with and without RBD when compared to HC.

The slope of the I/O curve by the influence of increasing TMS intensity was steeper in PD and multiple system atrophy (24–27). But we did not find this phenomenon in our patients with RBD; however, the baseline MEP amplitude was higher in TRA-RBD than in TRN-RBD and HC although it was not statistically significant (Figure 2). Lou et al. reported a higher baseline MEP in clinical PD. Valls-Sole et al. described the borderline larger baseline MEP amplitude at 100% rMT of TMS (24, 28). Cantello et al. also illustrated that the resting MEP amplitude was augmented in PD, but this enhancement would disappear when the MCV reached 100% (29). Therefore, it is intriguing to note the slope of the I/O curve after increasing the power of voluntary muscular contraction. Figure 3B identified that the MEP facilitation ratio in our patients with RBD would decrease significantly when the MCV is from baseline to 50% and 100%. One study in PD focused on the same issue, reporting that increasing the degree of voluntary muscle activation elicited a smaller increasing MEP area (24). The authors concluded that the control of the excitability of the motor system is abnormal in PD patients, with an enhancement of excitability at rest and weak energization during voluntary muscle activation. This concept also applied to our TRA-RBD patients. In our study, the baseline MEP amplitude in TRA-BRD was higher than in TRN-BRD. Still, the facilitatory effect of MEP amplitude by increasing the MCV was significantly decreased than in TRN-RBD by calculating the MEP ratio (Figure 3B); however, if comparing the difference of the absolute MEP amplitude, there would only be borderline significance (Figure 3A). Robert Chen et al. hypothesized that the activation-deactivation process of the motor cortex was impaired in PD. Thence, the depression of antagonists’ premovement facilitation was inadequate for satisfactory agonist contraction (30). From these viewpoints, measuring abnormalities in I/O curves in RBD patients through enhancing MVC instead of increasing the external TMS intensity might be more efficient in an investigative process. Moreover, evaluating the changes in the MEP ratio might be a more feasible parameter to detect impaired recruitment in the facilitation process.

The most consistent changes in cortical excitability in neurodegenerative diseases were the reduction of SICI and the shortening of contralateral SP. These abnormalities have not only been reported in PD but also in dystonia, Huntington’s chorea, and Sialidosis type I (31). We also identified the same abnormality in TRA-RBD but not in TRN-RBD. It is worth noting that if we pooled all patients with RBD together, the comparison with HC showed no significant difference, just like the previously published reports (15, 16). The SP was considered a mixture of spinal and intracortical inhibitory circuits mediated by the GABAergic mechanism. Inghilleri et al. proposed that spinal inhibition contributes to the first 50 ms of SP, and the latter portion, longer than 75 ms, was controlled by intracortical inhibition (32). In our study, the SP of HC was 120.1 ± 14.4, the SP of TRN-RBD was 109.8 ± 18.4, and the SP of TRA-RBD was 91.1 ± 20.0. We, therefore, believe that the shortening of SP in patients with RBD is mainly due to disinhibition in the motor cortex. Although only the TRA-RBD showed a significant decrease, the trend in the shortened SP may imply the TRA-RBD had more impaired intracortical inhibition of GABAergic effects while the TRN-RBD had a lesser tendency.

As noted in SP, we also demonstrated the transitions in intracortical inhibition and facilitation in a different extension (Table 2). The mean SICI was 53.4% in HC, 70.2% in TRN-RBD, and 98.6% in TRA-RBD. The SICI circuit is thought to be mediated mainly by GABA_A_ and is connected to disease severity (14). The degree of deficit in SICI was reported to be correlated with bradykinesia in PD and the Sleep Atonia Index in RBD (13, 16). Even though we did not recruit clinical PD in this study, the nearly absent SICI was comparable to the reports in the PD cohort (13, 33–37).

.It is interesting that in our study, the ICF was significantly increased in TRA-RBD. We found that the TRN-RBD had the highest ICF ratio, followed by TRA-RBD, then the HC. In clinical PD, the ICF showed no difference from HC. There were scarce reports describing ICF in patients with RBD (Table 3). Our finding was contrary to the study of Lanza et al. in idiopathic RBD and Bhattacharya et al. in patients with clinical PD comorbid with RBD. All three studies revealed abnormal ICF, not normal ICF, as found in PD. Why the TRN-RBD showed the highest ICF ratio and not the TRA-RBA whose striatal dopaminergic neuron degeneration should be more profound needs to be researched. We could not make any conclusion because our sample size was too small. We speculate the possible assumption by borrowing the concept established from the non-manifesting carriers of DYT1. Clinically, non-manifesting carriers of the DYT1 gene showed no dystonic symptoms, but the decreased intensity of SICI and ICF already existed (38). The cortical plasticity study using theta burst stimulation of TMS revealed hyperplasticity in non-manifesting carriers and hypoplasticity in manifesting carriers (39). Rothwell et al. suggested that cortical excitability might perform some kind of compensatory protection mechanism to prevent the manifestation of dystonic symptoms. If ICF in clinical PD showed no significant difference to HC, the enhanced ICF in RBD would possibly be a protective mechanism; therefore, TRN-RBD showed the highest ICF ratio to provide more protection. When the neurodegenerative process progressed, if any, the ICF ratio decreased and finally reached the level of HC and clinically manifesting PD. Lanza et al. reported that the ICF decreased by 30% in idiopathic RBD patients only, but the ICF decreased by 80% in PD patients with RBD. It could be possible that patients with RBD without manifested PD symptoms would have some mechanism to protect against the over-reduced glutamatergic transmission in clinically manifested PD. Of course, the assumption needs further elucidation and warrants a large-scale study with detailed protocols.

The clinical manifestation of Parkinson’s disease emerges when abnormal dopamine depletion generates profound alteration in the neural activity of cortico-basal ganglia motor loops (42). These alternations of unbalanced glutamatergic and GABAergic activities lead to the dysfunctional output of the cortical motor circuit (42). The neuropathology of RBD is primarily the degeneration of glutamatergic and GABAergic neurons in the sublaterodorsal nucleus (SLD) located at the low brain stem (43). Also, brain stem dysfunction was essential to the non-motor symptoms of clinical PD. It has been shown that clinical PD comorbid with RBD showed an increased tendency to develop faster cognitive decline, more axial symptoms, further sleep problems, and severer autonomic disturbances. Therefore, from the clinical point of view, in patients with RBD but without motor symptoms of PD, it could be possible that the degeneration process has been processed in the brain stem. This warrants careful evaluation if the other PD’s non-motor symptoms exist.

Research has shown that clinical PD patients with RBD had more profound inhibition of SICI and lower intensity of ICF than those without RBD (18). This finding might lead to the thought that additional and widespread electrophysiological deficits would present when clinical PD is comorbid with RBD. Although we did not recruit patients with clinical PD, our study found that TRA-RBD has similar cortical excitability alterations to that reported in clinical PD (Table 3). It is difficult to conclude that RBD patients with abnormal cortical excitability will surely evolve into clinical PD; however, it might be possible that, in pathophysiological terms, RBD patients with abnormal TRODAT-1 and cortical excitability carry a higher risk of prodromal PD.

## 5. Limitations

This study has some limitations. First, the sample size was not large enough to sufficiently reach statistical significance in some parameters of our results. We found a similarity between TRN-RBD and HC and a substantial difference between TRA-RBD and HC; however, the TRA-RBD and TRN-RBD did not have a significant difference. Nevertheless, this viewpoint warrants further large-scale study to elucidate the cortical excitability changes that we did not note in TRN-RBD. Second, our RBD patients were recruited by clinical diagnosis and questionnaire and not by polysomnography documenting the REM sleep without atonia, which is the revised diagnostic criteria suggested by the International Classification of Sleep Disorders. Third, we did not evaluate other non-motor symptoms in our cohort; therefore, the association of changes in cortical excitability with other non-motor symptoms in RBD needs further elucidation. Fourth, we did not recruit clinical PD patients in this study, and our study was not a long-term follow-up study. Therefore, we could not conclude that the cortical excitability alteration that would work as a biomarker to predict patients with clinical PD will be RBD.

## Data availability statement

The raw data supporting the conclusions of this article will be made available by the authors, without undue reservation.

## Ethics statement

The studies involving human participants were reviewed and approved by Chang Gung Memorial Hospital institutional review board. The patients/participants provided their written informed consent to participate in this study.

## Author contributions

R-SC contributed to conception and design of the study. S-CS and Y-YL performed the transcranial magnetic stimulation study. S-CS and Y-CC organized the database. R-SC, S-CS, Y-CC, and Y-HW analysed and interpreted the data. R-SC, S-CS, Y-CC, Y-HW, and JH drafted the manuscripts. All authors contributed to the article and approved the submitted version.

## Conflict of interest

The authors declare that the research was conducted in the absence of any commercial or financial relationships that could be construed as a potential conflict of interest.

## Publisher’s note

All claims expressed in this article are solely those of the authors and do not necessarily represent those of their affiliated organizations, or those of the publisher, the editors and the reviewers. Any product that may be evaluated in this article, or claim that may be made by its manufacturer, is not guaranteed or endorsed by the publisher.
